# Informed Consent Procedure for Research Including Patients with Parkinson's Disease and Cognitive Impairment

**DOI:** 10.1002/mdc3.70174

**Published:** 2025-06-09

**Authors:** Vibuthi Sisodia, Bart E.K.S. Swinnen, Evelien Lemstra, Gert J. Geurtsen, Albert F.G. Leentjens, Martin A.J.M. Buijsen, Maartje H.N. Schermer, Rob M.A. de Bie

**Affiliations:** ^1^ Amsterdam UMC, Amsterdam Neuroscience University of Amsterdam, Department of Neurology Amsterdam The Netherlands; ^2^ University Hospitals Leuven Department of Neurology Leuven Belgium; ^3^ Amsterdam UMC, Amsterdam Neuroscience Vrije Universiteit Amsterdam, Department of Neurology Amsterdam The Netherlands; ^4^ Amsterdam UMC, Amsterdam Neuroscience University of Amsterdam, Department of Medical Psychology Amsterdam The Netherlands; ^5^ Maastricht University Medical Center Department of Psychiatry Maastricht The Netherlands; ^6^ Erasmus School of Law and Erasmus School of Health Policy & Management Erasmus University Rotterdam Rotterdam The Netherlands; ^7^ Erasmus School of Health Policy & Management Erasmus University Rotterdam Rotterdam The Netherlands

**Keywords:** Parkinson's disease, cognitive impairment, informed consent, Parkinson's disease dementia

## Introduction

Motor and cognitive symptoms are frequent phenotypic presentations of various neurodegenerative disorders. These symptoms often coexist, leading to an intricate interplay between motor and cognitive functions. Cognitive symptoms are particularly frequent in movement disorders such as Parkinson's disease (PD), progressive supranuclear palsy, and Huntington's disease.^1^ In PD, 50% of patients develop mild cognitive impairment (MCI) within five years after diagnosis.^2^ The prevalence of Parkinson's disease dementia (PDD) is 20% after five years and increases to 50% after ten years.^3^ In general, cognitive symptoms in PD predominantly affect attention, working memory, executive function, and visuospatial abilities, with language and memory relatively preserved.^4^

PD patients with cognitive impairment, particularly late‐stage PD‐MCI and dementia, are rarely included in clinical research due to concerns over the capacity to provide informed consent and potential issues with compliance. Hence, many treatments routinely performed in clinical practice lack evidence‐based support when applied to cognitively impaired patients. While certain new treatments, such as oral medications, may be initiated regardless of cognitive status, advanced therapies in PD for the motor symptoms, such as deep brain stimulation (DBS), are considered contra‐indicated if concurrent cognitive impairment is present.^5^ There are concerns that after DBS PDD patients might experience insufficient improvement of motor symptoms and that DBS may even lead to accelerated cognitive decline. Yet, recent evidence does not support this.^6^, ^7^ Similarly, pump therapies, including apomorphine and levodopa pumps, have not been thoroughly studied in patients with PDD. Given that PD patients with cognitive impairment experience lower quality of life, research involving this population remains essential.^8^, ^9^ In addition, the degree of risk varies depending on the type of study. For instance, observational studies generally pose fewer risks concerning patient safety and compliance compared to interventional trials involving new treatments or invasive procedures. Insights from Alzheimer's disease research suggest that patients with dementia may retain the capacity to provide informed and voluntary consent.^10^ Therefore, modifications to the informed consent procedure for patients with late‐stage PD‐MCI and PDD are necessary. When considering advanced care planning in PD, it is important to properly assess the decision‐making capacity to accurately document patients’ wishes. Addressing these aspects within both research and clinical settings is crucial to ensure that decisions are made in an ethical manner and truly reflect the patient's preferences and values. In the informed consent process, assessing decision making capacity is a critical first step. Currently, there are no clear guidelines on how to assess decision making capacity or conduct the informed consent procedure regarding treatment options in patients with cognitive impairment.

We aim to address these concerns and provide practical guidance on obtaining informed consent for research in PD patients with cognitive impairment. First, the capacity to consent to research participation in general will be discussed, followed by an exploration of factors that can negatively affect the capacity to provide informed consent. Lastly, we will present practical considerations for obtaining consent for research including PD patients with cognitive impairment. This approach builds on the procedures of an ongoing randomized controlled trial involving deep brain stimulation in patients with PDD (DBS‐MODE).^11^ This guidance may also be implemented in routine clinical practice and could be applicable in other movement disorders with cognitive impairment as well.

### Capacity to Consent to Research Participation

In clinical research, informed consent procedures are pivotal for maintaining ethical standards.^12^ This entails ensuring that participants comprehend the nature of the study as well as treatment options outside of the clinical trial. To facilitate this, patients should be offered a reasonable amount of time with ample opportunity to ask questions regarding the study. Based on this, participants can make an informed and voluntary decision. Adequate informed consent capacity is required to perform these procedures appropriately. This capacity ensures that participants have the cognitive and volitional abilities necessary to make decisions about their participation in research studies. To assess the informed consent capacity, the Appelbaum and Grisso criteria can be applied.^13^ First, the participant must be able to comprehend the information about, but not limited to, the study's purpose, risk and benefits. Next, the participant should recognize how the provided information is relevant to their own situation and make a reasoned decision. Lastly, the participant must be able to communicate a clear and consistent decision regarding participation in the study.

### Factors Affecting the Capacity to Consent Including PD Patients with Cognitive Impairment

Participants with PD and cognitive impairment may have diminished capacity to provide informed consent. The extent and nature of the cognitive symptoms and their impact on the informed consent capacity can vary among patients.^14^ They may struggle to fully comprehend the presented information or be more susceptible to coercion. Patients with PD can experience distinct cognitive symptoms, which may specifically impact the informed consent capacity: (1) decreased attention and concentration may affect the ability to focus and comprehend the presented information, (2) difficulties with memory may impair the ability to retain and recall information related to the research study (eg, study procedures, potential risks, benefits), (3) impaired executive function may hamper complex decision‐making, evaluation of the consequences of their choices and abstract thinking (eg, understanding the randomization process), (4) challenges with visuospatial abilities may impact the understandings of visual aids such as diagrams, and (5) language impairment may hinder comprehension and communication skills with patients struggling to articulate their thoughts and decision regarding study participation. Moreover, cognitive impairment in PD is characterized by cognitive fluctuations, with patients experiencing periods of relative clarity mixed with periods of confusion. During periods of confusion, the patient's capacity to comprehend and make decisions may be compromised. In addition, the diminished capacity to provide informed consent can in part also be attributed to neuropsychiatric symptoms, such as impulsivity resulting from impulse control disorder or apathy. Decision‐making capacity in PD patients with cognitive impairment may also be influenced by medication‐induced fluctuations (eg, on‐medication phase, off‐medication phase, wearing off). During an on‐medication phase patients may be hypomanic and during an off‐medication phase patients might exhibit depressive feelings, fatigue or diminished alertness. These fluctuations could adversely affect their ability to engage effectively in the informed consent process, particularly in recognizing how the information is relevant to their own situation.

### Considerations for the Informed Consent Process Including PD Patients with Cognitive Impairment

For PD patients with normal cognition, the standard informed consent procedure may suffice (Fig. S1). The recommendations for the informed consent process in PD patients with cognitive impairment are summarized in Table 1. In patients with either confirmed or suspected cognitive impairment, the decision‐making capacity should be assessed as part of the informed consent procedure. In general, the criteria established by Appelbaum and Grosso can be applied.^13^ However, cognitive impairment may influence decision‐making capacity in PD patients. The use of the MacArthur Competence Assessment Tool provides a more structured approach and is therefore recommended for this purpose.^15^, ^16^, ^17^ This tool evaluates decision‐making capacity in patients with cognitive impairment by assessing understanding, appreciation, reasoning, and expression of choice.

**TABLE 1:** Recommendations for the informed consent procedure in PD patients with cognitive impairment

Assessment of decision‐making capacity
Use the MacArthur Competence Assessment Tool
Addressing cognitive symptoms in PD
Attention and concentration
Ensure patient is well rested
Schedule appointments in the early afternoon during an on‐medication period
Schedule sufficient time for appointments (e.g., 60 min)
Memory
Focus on key concepts of the study
Executive function
Provide step‐by‐step guidance
Use scenarios or examples for complex concepts
Visuospatial abilities
Use simple, clear illustrations using icons (avoid diagrams)
Present information in sequential manner
Language
Use simple language and short clear sentences (avoid medical jargon)
Involving caregivers
Emphasize that the patient's preferences and values remain central
Consider proxy consent for incompetent patients
Long lasting studies
Use advanced directives
Assess patients willingness to continue participation annually

Abbreviation: PD, Parkinson's disease.

For PD patients with cognitive impairment, it is necessary to address the specific challenges related to attention and concentration impairments. Given the impact of motor fluctuations on attention, appointments should be scheduled when patients are typically more alert, such as in the early afternoon during an on‐medication period. Ensuring patients are well‐rested before the appointment can also improve their engagement in the consent process. Additionally, longer appointments (eg, 60 min instead of 30 min) should be scheduled to ensure sufficient time for the various components of the informed consent conversation. For patients with executive function impairments, providing step‐by‐step guidance and using scenarios or examples can help clarify complex concepts like randomization. Spatial insight challenges can be addressed by employing simple, clear illustrations using icons rather than complex diagrams. Presenting information in a sequential manner can further facilitate understanding of the study. To optimize comprehension, the information should be communicated in simple language using short clear sentences, avoiding medical jargon. The conversation should focus on the key concepts of the study.

Caregivers could play a vital role in the informed consent process by assisting with information processing and asking questions on their behalf. However, it is crucial that caregivers are adequately equipped to perform this role. While caregiver involvement can enhance understanding and emotional support, it may also introduce challenges such as potential conflicts of interest and reduced patient autonomy due to paternalism and caregiver overreach. Safeguarding patient autonomy requires emphasizing to caregivers that their role is to ensure the patient's preferences and values remain central throughout the process.

#### Proxy Consent

At each stage of the informed consent process, the assessing physician can conclude that the patient lacks sufficient capacity to consent to participate in the study. Ideally, the assessing physician should not be the study physician to avoid conflicts of interest. Depending on the study's aim and design, there is legal provision to include incompetent patients.^18^ In such cases, the patient is either excluded or their legal representative must provide informed consent on their behalf (ie, proxy consent). Careful consideration must be given to ensure that the proxy is acting in accordance with the patient's presumed wishes and is not influenced by their own biases or interests. Proxy consent is acceptable only if there is a reasonable expectation that the patient would benefit from participation. If this expectation is not met, participation should be avoided. Additionally, if the patient actively refuses to participate, even with proxy consent, inclusion is not possible.^18^

#### Advanced Directives and Periodic Confirmation of Consent in Long Lasting Studies

Patients capable of consent may formulate advanced directives to specify their wishes regarding future participation in research if they lose the ability to provide informed consent. This may provide reassurance to caregivers regarding the patient's involvement in research. Alternatively, if advanced directives are not available or applicable, proxy consent may be utilized. Similar to the informed consent procedure at baseline, if the participant actively refuses to continue at any point even with proxy consent, their decision to withdraw should be respected.^18^

In long‐term or burdensome studies, it is essential to periodically, for instance annually, revisit the research context with participants. The aim is to clarify which assessments and procedures are conducted as part of the study and which are part of routine clinical care, followed by an evaluation of the patient's willingness to continue participation. This approach helps ensure that patients remain adequately informed and capable of making an ongoing, voluntary decision about their involvement.

#### Recommendations for Research on Informed Consent Capacity

The recommendations provided in this manuscript are based on existing research on Alzheimer's disease and have been adapted to the specific clinical profile of PD patients (eg, by incorporating guidance on how to account for fluctuations in attention (Table 1)).^10^ Given the challenges of obtaining informed consent from patients with PDD in clinical trials and the lack of evidence or guidance on this topic, future research should evaluate both the patient's and caregivers’ experience with this process. Their feedback and suggestions can be used to enhance the procedure. Moreover, the informed consent process itself could be the focus of future qualitative and experiential research. This could help establish an evidence based ethical framework for obtaining informed consent from patients with cognitive decline.

## Conclusion

Cognitive impairment profoundly affects the quality of life in PD, highlighting the critical need for research in this population. Special attention must be given to the informed consent capacity to safeguard their rights and well‐being. Key considerations for obtaining informed consent from PD patients with cognitive impairment include using appropriate assessment tools, adapting the informed consent process to the cognitive profile of PD, and involving caregivers.

## Author Roles

(1) Research project: A. Conception, B. Organization, C. Execution. (2) Statistical Analysis: A. Design, B. Execution, C. Review and Critique. (3) Manuscript: A. Writing of the first draft, B. Review and Critique.

V.S.: 3A, 3B

B.E.K.S.S.: 3A, 3B

A.W.L.: 3B

G.J.G.: 3B

A.F.G.L.: 3B

M.A.J.M.B.: 3B

M.H.N.S.: 3B

R.M.A.B.: 3A, 3B

## Disclosures

**Ethical Compliance Statement:** The authors confirm that the approval of an institutional review board was not required for this work. No informed consent was obtained. We confirm that we have read the Journal's position on issues involved in ethical publication and affirm that this work is consistent with those guidelines.

**Funding Sources and Conflict of Interest:** No specific funding was received for this work. The authors declare that there are no conflicts of interest relevant to this work.

**Financial Disclosures for the Previous 12 Months:** RB reports research grants from ZonMw (Netherlands governmental fund for health research), Stichting ParkinsonFonds (charitable foundation), Parkinson Vereniging (Netherlands patient organization), ParkinsonNL (charitable foundation), and Medtronic all paid to the institution. All other authors declare that there are no additional disclosures to report.

## Supporting information


**Supplementary Figure S1.** Standard informed consent procedure for research in PD patients with normal cognition.

## Data Availability

Data sharing not applicable to this article as no datasets were generated or analysed during the current study.
