# Supplementary material for: Informed Consent Procedure for Research Including Patients with Parkinson's Disease and Cognitive Impairment
Source: Mov Disord Clin Pract. 2025 Jun 9;12(10):1507–10. doi: 10.1002/mdc3.70174 (PMC12528961; doi:10.1002/mdc3.70174)
Supplement: Supplementary file 1 — Supplementary Figure S1. Standard informed consent procedure for research in PD patients with normal cognition. [file MDC3-12-1507-s001.docx]

Data Supplement

**Informed consent procedure for research including patients with Parkinson’s disease and cognitive impairment**

V.Sisodia MD^1^, B.E.K.S. Swinnen MD PhD^1,2^, A.W. Lemstra MD PhD^3^, Gert J. Geurtsen PhD^4^, Albert F.G. Leentjens MD PhD^5^, Martin A.J.M. Buijsen PhD^6^, Maartje H.N. Schermer MD PhD^7^, Rob M.A. de Bie MD PhD^1^

**
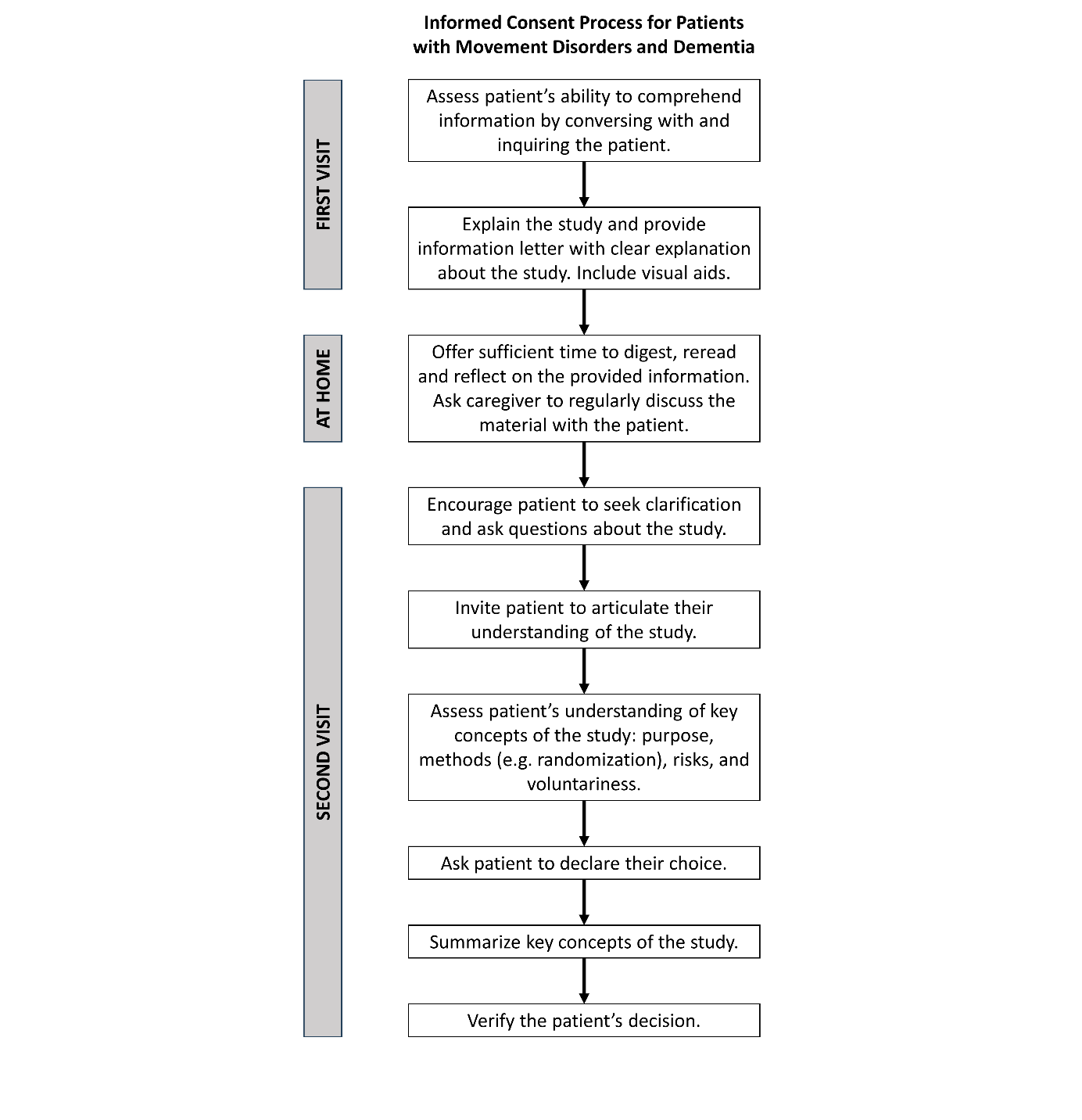
**

**Supplementary Figure 1**. Standard informed consent procedure for research in PD patients with normal cognition.
